# Synthesis, Characterization, and Evaluation of a Hindered Phenol-Linked Benzophenone Hybrid Compound as a Potential Polymer Anti-Aging Agent

**DOI:** 10.3390/antiox13080894

**Published:** 2024-07-24

**Authors:** Shenshuai Wang, Yingjie Huang, Weiye Sun, Xufeng Lin

**Affiliations:** Department of Chemistry, Zhejiang University, Hangzhou 310058, China

**Keywords:** anti-aging agent, hindered phenol, antioxidant, benzophenone, UV absorber

## Abstract

Hindered phenol antioxidants and benzophenone UV absorbers are common polymer additives and often used in combination applications to enhance the anti-aging performance of polymer materials. This study primarily aims to incorporate hindered phenol and benzophenone structures into a single molecule to develop a multifunctional polymer additive with good anti-aging performance. Thus, a novel potential polymer anti-aging agent, namely 3-(3,5-di-tert-butyl-4-hydroxyphenyl)propionic acid 3-(4-benzoyl-3-hydroxyphenoxy)propyl ester (**3C**), was synthesized using 3-(3,5-di-tert-butyl-4-hydroxyphenyl)propionic acid, 3-bromo-1-propanol, and 2,4-dihydroxybenzophenone as raw materials by two-step procedure. The structure of compound **3C** was characterized by nuclear magnetic resonance (NMR), high-resolution mass spectrometry (HRMS), Fourier-transform infrared (FT-IR) spectroscopy, and X-ray single crystal diffraction. Its thermal stability and UV resistance were assessed using thermogravimetric analysis (TGA) and UV absorption spectroscopy (UV). The compound **3C** as an additive was incorporated into the preparation of polyolefin elastomer (POE) films. The anti-aging performance of POE films was evaluated by measuring parameters such as oxidation induction time, melt flow index, transmittance, and infrared spectra of the artificially aged POE films. The results indicate that the compound **3C** exhibits a promising anti-aging performance in both thermo-oxidative aging and ultraviolet aging tests of POE films and is a potential polymer anti-aging agent.

## 1. Introduction

Polymer materials are widely used in packaging, construction materials, and devices. With the advancement of technology, new products are continually emerging, making significant progress in aspects such as being lightweight, high strength, and high transparency. The demand for polymer materials is expected to increase continuously. Fields such as new energy and electronic information are anticipated to become major growth drivers in the future polymer materials market.

Polymer materials used outdoors are susceptible to aging and degradation due to exposure to heat, oxygen, and light. During this process, oxidation structures such as carboxyl and peroxide groups form in the molecular chains, thereby reducing the resin’s anti-aging properties. The primary cause of polymer aging under natural light is UV radiation in the 290–400 nm range. For example, polypropylene is particularly sensitive to UV light with a wavelength of 310 nm due to the matching of polypropylene’s chemical bond energy with the energy of 310 nm UV light [1]. Consequently, polypropylene absorbs this UV light, leading to chemical bond breakage [2]. Oxygen combines at the polymer molecular chain breakage points to form peroxide radicals, which further disrupt the polymer structure through radical chain reactions. Elevated temperatures accelerate this aging process. As the application fields of polymer materials continue to expand, the requirements for their anti-aging properties are becoming increasingly stringent. Consequently, there is a growing demand for more effective polymer anti-aging additives.

Currently, commercial-grade polymer materials typically contain additives such as antioxidants and ultraviolet (UV) absorbers. The incorporation of these anti-aging additives endows polymer materials with resistance to biodegradation and photo-thermal oxidation, enabling them to withstand harsh outdoor environments over extended periods.

Ultraviolet (UV) absorbers are substances capable of selectively absorbing high-energy UV light and converting it into heat or harmless low-energy radiation, thereby releasing or dissipating the energy [3]. UV absorbers are widely used in polymer materials, textile processing, and other fields [4]. The common UV absorbers can be categorized into the six following major classes: salicylates, benzophenones, benzotriazoles, hindered amines, substituted acrylonitriles, and triazines [5,6,7]. Among these, benzophenones are the most one of commonly used UV absorbers. Benzophenone UV absorbers, such as UV-0, UV-9, and UV-531, are a typical example. These compounds contain two benzene rings connected by a carbonyl group, which endows them with a wide absorption wavelength range (290–400 nm), strong absorption capacity, good photostability, and low cost [8,9,10].

Hindered phenol antioxidants are compounds featuring a phenolic hydroxyl group on a benzene ring with substituent groups on one or both sides [11], often including two tert-butyl groups [12]. The spatial hindrance around the hydroxyl group facilitates the easy detachment of the hydrogen atom from the molecular structure. This process allows the antioxidant to donate a proton and react with peroxy radicals, alkyl radicals, and hydroxyl radicals, thereby deactivating these radicals and terminating the oxidation reaction. Common hindered phenol antioxidants include Antioxidant 1010 and Antioxidant 1076. These antioxidants are widely used due to their low toxicity and good compatibility, making them one of the most prevalent antioxidants in the market [13].

Due to the complexity of polymer systems, there are multiple factors affecting their anti-aging performance, such as the ability to terminate free radicals, compatibility with materials, and migration within the material [14]. To enhance the anti-aging performance of polymer materials, the combined use of UV absorbers and antioxidants is currently a primary solution [15]. However, due to the varying degrees of polarity among different antioxidants and UV absorbers, their compatibility is often poor. Achieving a synergistic effect typically requires combining the antioxidants [16] and UV absorbers with the polymer melt at high temperatures. Consequently, developing multifunctional polymer additives with various functional groups is a promising solution to address these compatibility issues and improve the overall performance of the materials.

This study primarily aims to incorporate benzophenone and hindered phenol structures into a single molecule as a hindered phenol-linked benzophenone hybrid compound bearing the antioxidant and UV absorber groups to develop a multifunctional polymer additive with good anti-aging performance. Thus, the novel compound 3-(3,5-di-tert-butyl-4-hydroxyphenyl)propionic acid 3-(4-benzoyl-3-hydroxyphenoxy)propyl ester (**3C**) were synthesized from 3-(3,5-di-tert-butyl-4-hydroxyphenyl)propionic acid, 3-bromo-1-propanol and 2,4-dihydroxybenzophenone by a two-step procedure. The structure of this compound **3C** was characterized using nuclear magnetic resonance (NMR) spectroscopy, high-resolution mass spectrometry (HRMS), Fourier-transform infrared (FT-IR) spectroscopy, and X-ray single crystal diffraction. Its thermal stability and UV resistance were assessed using thermogravimetric analysis (TGA) and UV absorption spectroscopy. Subsequently, the compound **3C** as an additive was incorporated into the preparation of polyolefin elastomer (POE) films and evaluated for its effect on the anti-aging performance of POE films. The POE films were crosslinked using dicumyl peroxide (DCP) and triallyl isocyanurate (TAIC) as crosslinking agents. The anti-aging performance of POE films was evaluated by measuring parameters such as oxidation induction time, melt flow index, transmittance, and infrared spectra of the artificially aged POE films. 

Due to concerns over the safety of benzophenone derivatives, several countries have begun to impose stricter regulations and reassess the maximum permissible levels of these additives in commercial products. Although 2,4-dihydroxybenzophenone (UV-0), is used as a UV absorber in cosmetics and plastics [17,18], some studies have indicated that benzophenone derivatives may exhibit certain toxicities in humans, including endocrine disruption and adverse effects on reproductive and genetic health [19]. Thus, as a polymer anti-aging agent with potential applications, the sanitary safety of 3C need more experimental evaluation in future. 

## 2. Materials and Methods

### 2.1. Reagents and Instruments

POE: ENGAGE™ 8450 Polyolefin Elastomer, manufactured by The Dow Chemical Company (Midland, MI, USA);

DCP: Supplied by Shanghai Aladdin Bio-Chem Technology Co., Ltd. (Shanghai, China);

TAIC: Supplied by Shanghai Dipu Biotechnology Co., Ltd. (Shanghai, China);

3-(3,5-di-tert-butyl-4-hydroxyphenyl) propionic acid: Industrial grade;

Thionyl chloride: Industrial grade;

Triethylamine: Industrial grade;

Potassium carbonate: Industrial grade;

3-Bromo-1-propanol: Industrial grade;

2,4-Dihydroxybenzophenone: Industrial grade.

### 2.2. Synthetic Methods and Preparation Processes

The synthesis of the compound 3-(3,5-di-tert-butyl-4-hydroxyphenyl) propionic acid-3-(4-benzoyl-3-hydroxyphenyl) propyl ester (**3C**) proceeded as follows: To a dry 500 mL single-neck flask, 3-(3,5-di-tert-butyl-4-hydroxyphenyl)propionic acid (5.572 g, 20 mmol) was added and dissolved in 100 mL of dichloromethane. Thionyl chloride (2.2 mL, 30 mmol) was then added dropwise under continuous stirring in an ice-water bath. Upon completion of the addition, the reaction mixture was heated to 40 °C and maintained at this temperature for 4 h. Following the reaction, the mixture was subjected to rotary evaporation at 40 °C to remove any unreacted thionyl chloride, yielding 3-(3,5-di-tert-butyl-4-hydroxyphenyl)propanoyl chloride, which did not require further purification.

**Figure 1:**
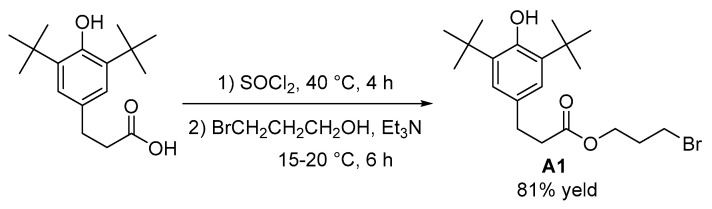
Synthesis of intermediate **A1**.

The obtained 3-(3,5-di-tert-butyl-4-hydroxyphenyl)propionyl chloride was dissolved in 100 mL of dichloromethane. To this solution, 3-bromo-1-propanol (4.30 g, 30 mmol) and triethylamine (2.8 mL, 20 mmol) were added, maintaining the temperature at 15–20 °C for 6 h. The reaction mixture was washed with water and separated. The organic phase was dried over anhydrous magnesium sulfate and the solvent was removed by rotary evaporation at 40 °C. The residue was purified by silica gel column chromatography (ethyl acetate/petroleum ether, 1/30, *v*/*v*), yielding a pale yellow oily liquid (6.427 g) identified as 3-(3,5-di-tert-butyl-4-hydroxyphenyl)propionic acid 3-bromopropyl ester (**A1**) with a yield of 81%. The synthesis route of intermediate **A1** is shown in Figure 1. 

**Figure 2:**
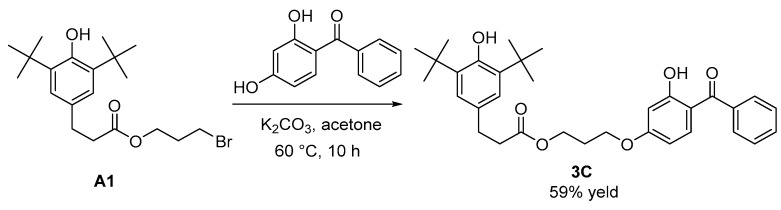
Synthesis of **3C**.

Into a dried 500 mL single-neck flask, 3-(3,5-di-tert-butyl-4-hydroxyphenyl)propionic acid 3-bromopropyl ester (3.596 g, 9 mmol) was added and dissolved in 100 mL of acetone. With stirring at room temperature, 2,4-dihydroxybenzophenone (2.921 g, 12 mmol) and potassium carbonate (1.878 g, 13 mmol) were added. The mixture was heated to 60 °C and refluxed for 10 h. Upon completion of the reaction, the solvent was removed by rotary evaporation at 40 °C. The residue was dissolved in 100 mL of ethyl acetate, washed with water, and the layers were separated. The organic phase was dried over anhydrous magnesium sulfate, and the solvent was removed by rotary evaporation at 40 °C. The product was purified by silica gel column chromatography (ethyl acetate/petroleum ether, 1/30, *v*/*v*), yielding a pale pink solid (2.810 g) identified as 3-(3,5-di-tert-butyl-4-hydroxyphenyl)propionic acid 3-(4-benzoyl-3-hydroxyphenoxy)propyl ester (**3C**) with a yield of 59%. The synthesis procedure of compound **3C** is shown in Figure 2.

Extrusion Process: Antioxidant (1 g), calcium stearate (2 g), and 1000 g of POE pellets were homogeneously mixed in a high-speed mixer at room temperature for 3 min. The mixture was then subjected to extrusion, drawing, and pelletization using a HAAKE Polylab OS torque rheometer (Thermo Electron (Karlsruhe) GmbH, Karlsruhe, Germany) equipped with a Rheomex PTW16/40 OS parallel co-rotating twin-screw extrusion unit. The main screw rotation speed was set at 300 rpm, with extruder zone temperatures set at 185 °C, 210 °C, 220 °C, and 185 °C. The extrusion process was repeated three times, and the resulting samples were dried in an oven at 80 °C for 4 h.

Preparation of POE Films: The HAAKE Polylab OS torque rheometer was preheated to 90 °C, operating at a rotation speed of 75 r/min, and charged with 50 g of POE pellets. Upon achieving a stable torque curve, 0.3 g of DCP and 0.25 g of TAIC were added and mixed for 3 min. Subsequently, 0.1 g of the anti-aging agent (omitted in the control group) was introduced, and the mixture was discharged post-uniform dispersion of additives. The resulting material was sheeted using an SY-6215-A2 two-roll mill (Dongguan Shiyan Precision Equipment Co., Ltd., Dongguan, China) and cut into samples. These samples were then placed into a mold measuring 75 × 60 × 0.5 mm^3^ and subjected to hot pressing at 135 °C for 25 min using a GT-7014-A50C laboratory press (Gotech Testing Machines (Dong Guan) Co., Ltd., Dongguan, China), followed by 10 min of cold pressing at room temperature. The resulting formed films were subsequently retrieved for further analysis.

### 2.3. Characterization of Compound Structure

The structure identification of compound **3C** and its synthetic intermediates was performed using HRMS, NMR, FT-IR, or X-ray single-crystal diffraction data. 

The chemical bond information of the compounds was recorded from a Nicolet iS10 FT-IR spectrometer (Thermo Fisher Scientific, Waltham, MA, USA) using ATR with the wavenumber range of 400–4000 cm^−1^.

The mass spectra of compound **3C** and its synthetic intermediate **A1** were obtained using an Agilent 6545 Q-TOF mass spectrometer (Agilent, Waldbronn, Germany) with Electron Spray Ionization (ESI).

^1^H NMR and ^13^C NMR spectra were recorded at 25 °C on a Bruker AVANCE 400M superconducting NMR spectrometer, with deuterated chloroform as the lock solvent. The working solution concentration for NMR was 30 mg/mL.

Single crystals of compound **3C** were obtained by solvent evaporation at a low temperature of 5 °C. The single-crystal structure of **3C** was determined using a Bruker D8 Venture Ims3.0 X-ray single-crystal diffractometer (Bruker, Berlin, Germany).

The thermogravimetric analysis (TGA) of compound **3C** was conducted using a DSC Q100 thermal analysis system (TA Instruments, New Castle, DE, USA). The TGA test was performed under a nitrogen atmosphere, heating from 20 °C to 600 °C at a rate of 10 °C·min^−1^, with weight–temperature curves recorded. The differential scanning calorimetry (DSC) test was performed in the temperature range of 20–200 °C, at a heating rate of 10 °C·min^−1^, with heat flow–temperature curves recorded.

The UV absorption spectrum of compound **3C** was measured using a UV-2600 UV-Vis spectrophotometer (Shimadzu Co., Ltd., Kyoto, Japan). The test sample was dissolved in methanol at a concentration of 0.0001 mol·L^−1^, with a wavelength scan range of 200–500 nm.

### 2.4. Aging Test and Performance Characterization

Thermal oxidative aging experiment: The specimens were subjected to thermal oxidative aging within a controlled environment chamber set at a constant temperature of 85 °C and relative humidity of 85%. Each aging cycle lasted for 120 h, with a total continuous aging duration of 600 h.

UV aging experiment: UV aging tests were conducted using a UV-irradiation weathering tester equipped with UV-A340 lamps, operating at an irradiance of 150 W/m^2^ and a temperature of 60 °C. Each aging cycle lasted for 200 h, with a total continuous aging duration of 400 h.

UV absorption capacity test of POE film: Utilize UV-2100 to measure the ultraviolet absorption curve of POE film within the wavelength range of 200–380 nm, with transmittance plotted on the vertical axis.

DSC oxidative induction time test: A 5 mg sample was subjected to rapid heating under nitrogen protection, with the temperature ramped from 40 °C to 200 °C at a rate of 20 °C/min. Upon reaching 200 °C, the temperature was maintained for 5 min. Subsequently, the atmosphere was switched to oxygen (at a flow rate of 50 mL/min) until a discernible exothermic oxidation reaction occurred, at which point the test was terminated. The thermal flux–time curve of the process was meticulously recorded throughout the experiment.

Melt flow index measurement: In accordance with the specifications outlined in GB/T 3682.1-2018 standard [20], the melt flow rate of the samples was determined. The extrusion temperature was set at 230 °C, with a load of 2.16 kg, and a nozzle diameter of 2.095 mm was utilized.

Measurement of light transmittance: Following the guidelines outlined in the GB/T 2410-2008 standard [21], the transmittance curve of the POE film within the wavelength range of 380 nm to 110 nm was assessed using the UV-2100 instrument (UNICO, Shanghai, China). The average transmittance values were determined at wavelengths of 555 nm, 700 nm, and 900 nm.

Infrared spectral analysis: The structural changes of the POE film before and after aging were investigated using attenuated total reflection (ATR) spectroscopy within the spectral range of 4000 to 400 cm^−1^. The alterations in absorbance of functional groups during the aging process were tracked by examining the infrared spectra obtained before and after aging. The carbonyl index (*CI*) was calculated using the following formula:

$$CI=\frac{A}{A_{r}}$$

where

A represents the absorbance at the wavenumber of 1710 cm^−1^.

$A_{r}$ denotes the absorbance of the standard peak. In this study, the peak at 2920 cm^−1^ was designated as the reference peak.

## 3. Results and Discussion

### 3.1. Characterization of Products

**Figure A1:**
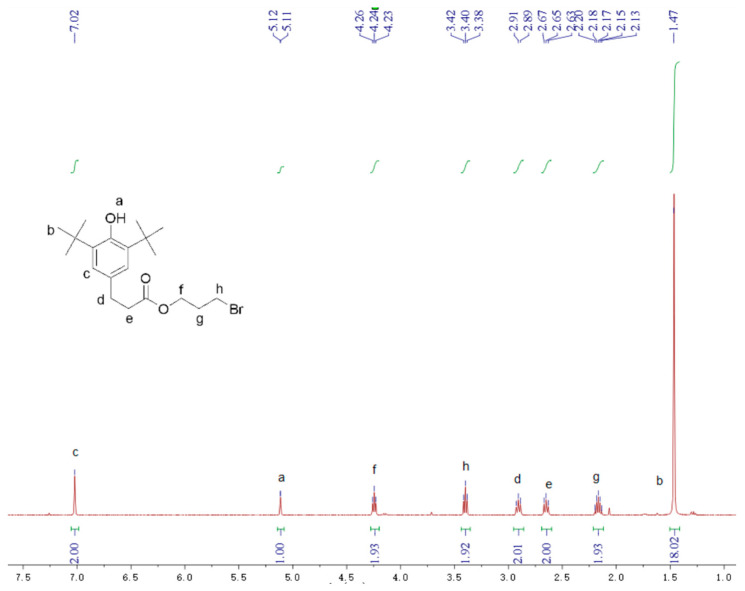
^1^H NMR spectra of compound **A1**.

**Figure A2:**
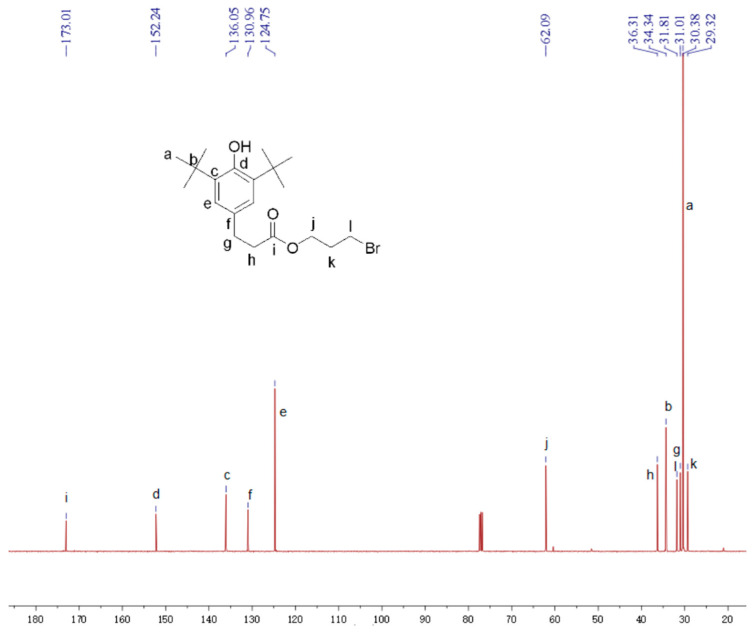
^13^C NMR spectra of compound **A1**.

The characterization data of **A1** are as follows: 

^1^H NMR (CDCl_3_, 400 MHz) δ = 7.02 (s, 2H), 5.11 (d, *J* = 1.1 Hz, 1H), 4.24 (t, *J* = 6.0 Hz, 2H), 3.40 (t, *J* = 6.5 Hz, 2H), 2.91 (t, *J* = 7.9 Hz, 2H), 2.65 (t, *J* = 7.8 Hz, 2H), 2.17 (p, *J* = 6.3 Hz, 2H), 1.47 (s, 18H); ^13^C NMR (CDCl_3_, 101 MHz) δ = 173.0, 152.2, 136.1, 131.0, 124.8, 62.1, 36.3, 34.3, 31.8, 31.0, 30.4, 29.3; FT-IR (film): *γ* = 571, 769, 875, 1001, 1027, 1056, 1087, 1122, 1159, 1234, 1311, 1360, 1392, 1435, 1733, 2341, 2361, 2872, 2915, 2958, 3067, 3638 cm^−1^; HRMS (ESI): calcd. for C_20_H_31_BrO_3_ [M-H]^−^ 397.1384, found 397.1380. NMR spectra of **A1** can be found in the Appendix A (Figure A1 and Figure A2). 

**Figure A3:**
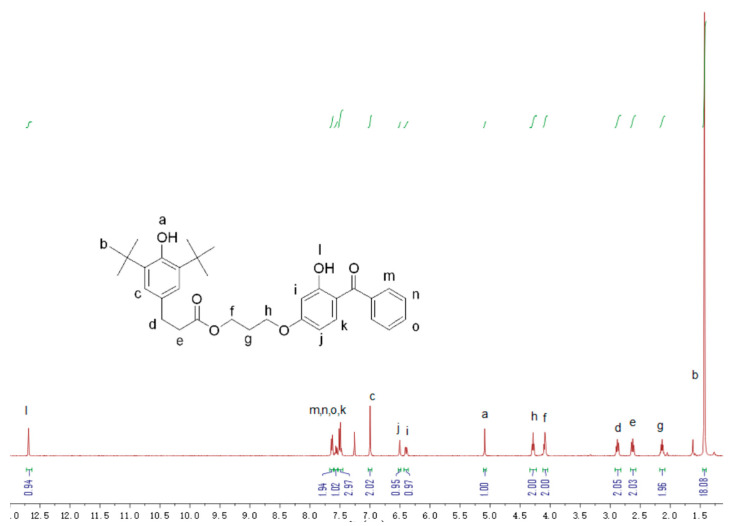
^1^H NMR spectra of compound **3C**.

**Figure A4:**
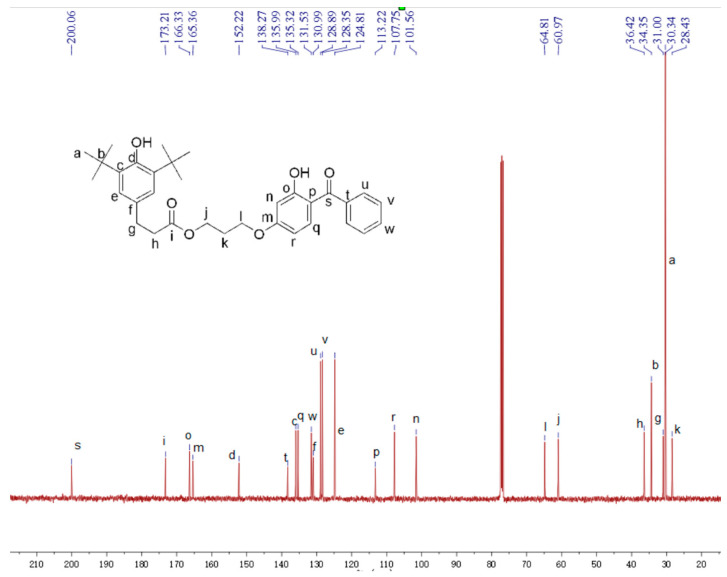
^13^C NMR spectra of compound **3C**.

The characterization data of **3C** are as follows: 

^1^H NMR (CDCl_3_, 400 MHz) δ = 12.69 (s, 1H), 7.63 (d, *J* = 7.1 Hz, 2H), 7.57 (t, *J* = 7.3 Hz, 1H), 7.49 (t, *J* = 7.5 Hz, 3H), 7.00 (s, 2H), 6.51 (d, *J* = 2.3 Hz, 1H), 6.40 (dd, *J* = 9.0, 2.4 Hz, 1H), 5.09 (s, 1H), 4.28 (t, *J* = 6.2 Hz, 2H), 4.09 (t, *J* = 6.1 Hz, 2H), 2.92–2.84 (m, 2H), 2.66–2.59 (m, 2H), 2.14 (p, *J* = 6.1 Hz, 2H), 1.43 (s, 18H); ^13^C NMR (101 MHz, CDCl_3_) δ = 200.06, 173.21, 166.33, 165.36, 152.22, 138.27, 135.99, 135.32, 131.53, 130.99, 128.89, 128.35, 124.81, 113.22, 107.75, 101.56, 64.81, 60.97, 36.42, 34.35, 31.00, 30.34, 28.43; FT-IR (film): *γ* = 628, 700, 749, 766, 1053, 1116, 1165, 1194, 1231, 1260, 1343, 1435, 1509, 1578, 1621, 1736, 2344, 2373, 2872, 2958, 3064 cm^−1^; HRMS (ESI): calcd. for C_33_H_40_O_6_ [M-H]^−^ 531.2752, found 531.2753. NMR spectra of **3C** can be found in the Appendix A (Figure A3 and Figure A4). 

**Figure 3:**
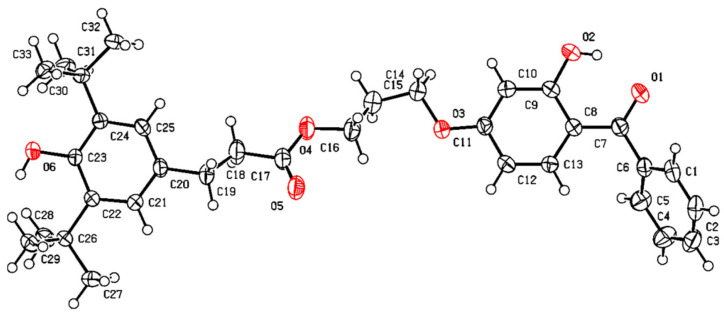
The X-ray single crystal diffraction pattern of compound **3C**.

X-ray single crystal diffraction [22]: The X-ray single crystal diffraction pattern of **3C** is shown in Figure 3. Space Group: P21, Cell: *a* 10.934 Å *b* 9.91 Å *c* 13.262 Å, *α* 90° *β* 92.227(2)° *γ* 90°.

**Figure 4:**
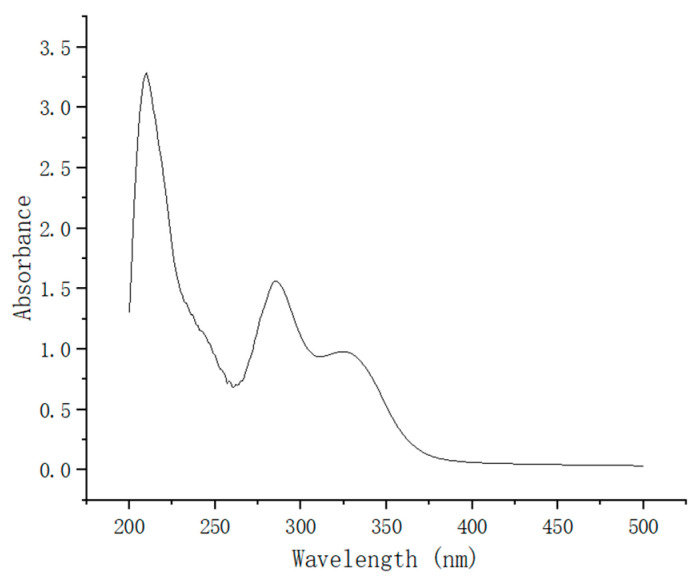
The UV absorption spectrum of compound **3C**.

Figure 4 depicts the UV absorption spectrum of compound **3C**, revealing prominent absorption peaks in the wavelength range of 260–350 nm. This observation suggests that **3C** exhibits effective absorption of ultraviolet radiation within this wavelength range.

**Figure 5:**
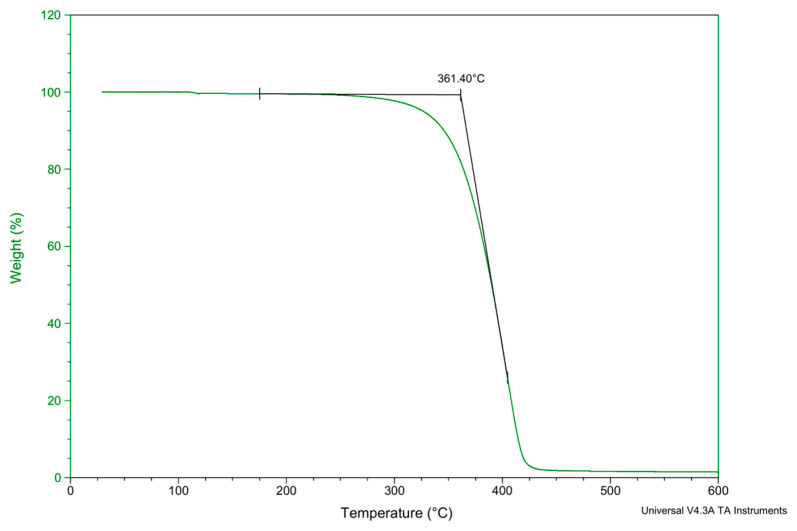
TGA test curve of compound **3C**.

The thermogravimetric analysis (TGA) test curve for **3C** is shown in Figure 5. It can be observed that compound **3C** exhibits significant mass loss only after reaching 360 °C. This indicates its excellent thermal stability, making it suitable as an anti-aging additive for polymers processed at high temperatures. Its applicability is, therefore, broad.

**Figure 6:**
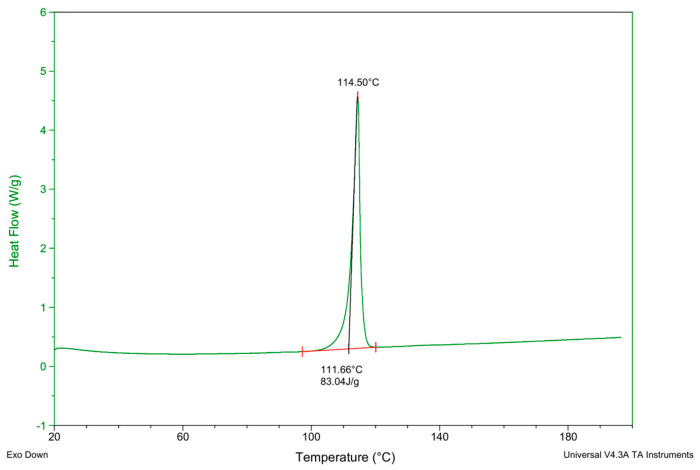
The DSC test curve of compound **3C**.

The DSC test curve for **3C** is depicted in Figure 6. It is evident that compound **3C** exhibits a melting point around 111 °C.

### 3.2. The Results of POE Aging Resistance Performance Testing

#### 3.2.1. The Impact of Different Anti-Aging Agents on the Oxidative Induction Time of POE

**Figure 7:**
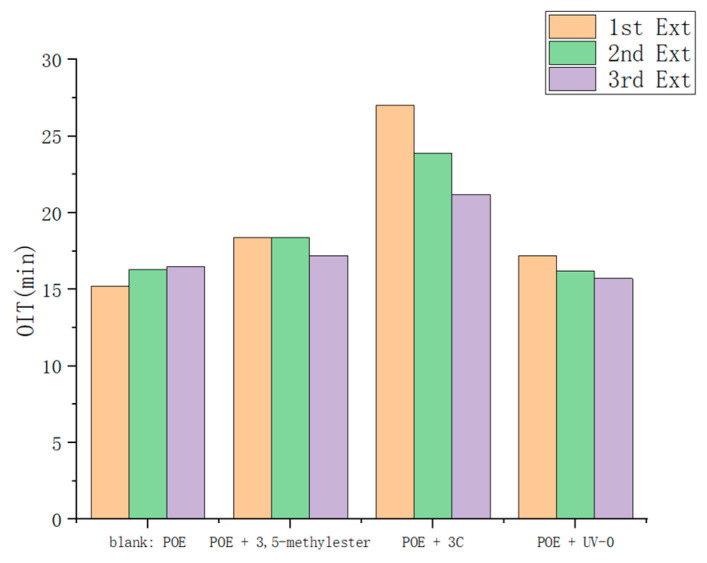
Oxidative induction time of POE pellets after multiple extrusions.

The oxidation induction time (OIT) of polymers provides a direct indication of material aging. As depicted in Figure 7, during the initial extrusion process, the POE samples supplemented with **3C** exhibited a significantly extended OIT (27 min) compared to the blank POE group (15 min), the POE group added with 3-(3,5-di-tert-butyl-4-hydroxyphenyl) propionic acid methyl ester (referred to as 3,5-methylester) (18 min), and the POE group added with UV-0 (17 min). With successive extrusion cycles, the thermal oxidative aging of POE intensified. By the third extrusion, the OIT of POE samples with **3C** declined to 21 min, still surpassing that of the blank group (16 min), the 3,5-methylester group (17 min), and the UV-0 group (15 min), underscoring the efficacy of **3C** in bolstering the thermal oxidative aging resistance of POE. 

#### 3.2.2. UV Transmittance of POE Films

**Figure 8:**
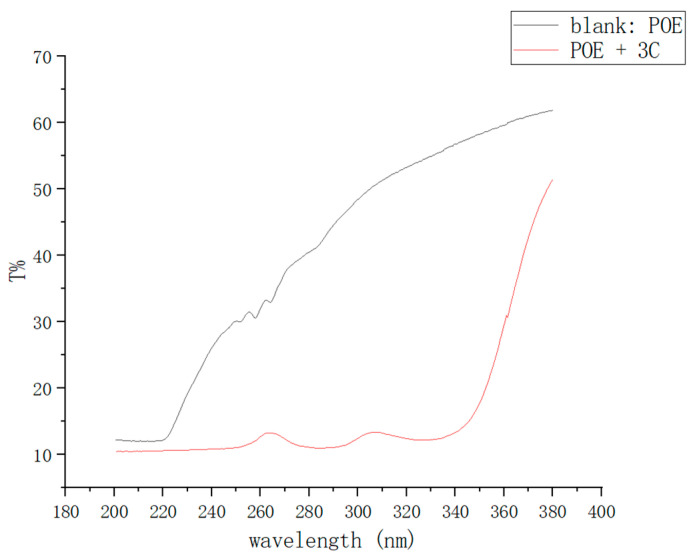
UV transmittance graphs of POE films.

As shown in the Figure 8, for the POE film without **3C** additive, the transmittance of UV light in the wavelength range of 280–340 nm is between 40% and 55%. In contrast, the POE film with **3C** additive exhibits a transmittance of only 10% within the corresponding wavelength range. This indicates that the POE film with **3C** additive absorbs the majority of UV light in the 280–340 nm range. Compound **3C** demonstrates excellent UV absorption capability within the POE film.

#### 3.2.3. The Impact of Different Anti-Aging Agents on the Melt Flow Index of POE

The impact of various anti-aging agents on the melt flow index (MFI) of POE is depicted in Table 1. The incorporation of **3C** into POE resulted in a significantly lower MFI (4.00 g/10 min) compared to both the blank group (4.80 g/10 min) and the 3,5-methylester group (4.64 g/10 min). In the absence of anti-aging agents to hinder oxidation reactions, the polymer chains experience oxidative degradation under the influence of heating and physical shear. Alkoxy radicals undergo β-scission, leading to the formation of ketones and alkyl radicals [23], consequently causing a reduction in molecular weight. This leads to enhanced flowability of the resin in the molten state, resulting in a higher melt flow rate [24]. Conversely, the addition of anti-aging agents to POE samples resulted in a lower melt flow rate, indicating the effective thermal stabilization role of these agents during the extrusion process.

#### 3.2.4. Light Transmittance Measurement of POE Films after Thermal Oxidative Aging

**Figure 9:**
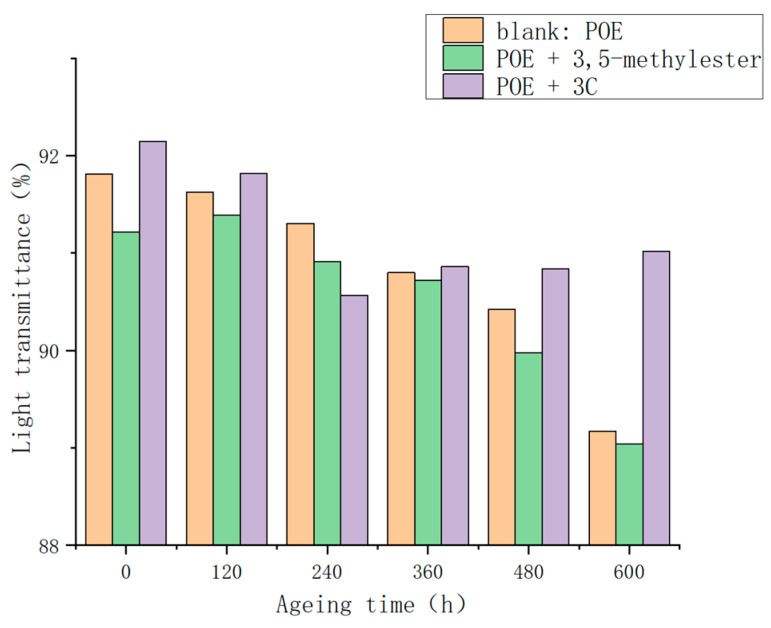
Light transmittance in POE films after thermal oxidative aging.

Following thermal oxidative aging at 85 °C for 600 h, the light transmittance of the POE film exhibited a declining trend (Figure 9). This phenomenon can be ascribed to two primary factors. Firstly, the film underwent the formation of chromophoric groups due to the combined effects of heat and oxygen, resulting in diminished light transmittance. Secondly, the non-crosslinked portion of the film underwent crystallization induced by heat, leading to the formation of larger-sized crystals and further reducing light transmittance. Both the blank group and the 3,5-methylester group demonstrated a noticeable decrease in light transmittance, dropping from 91% to 89%. Conversely, the POE film containing added **3C** maintained a light transmittance of over 90% after 600 h of thermal oxidative aging. This observation indicates the effective inhibition of rapid light transmittance decline by the self-made anti-aging agent **3C** against thermal oxidative aging.

#### 3.2.5. Carbonyl Index Determination in POE Film after Thermal Oxidative Aging

**Figure 10:**
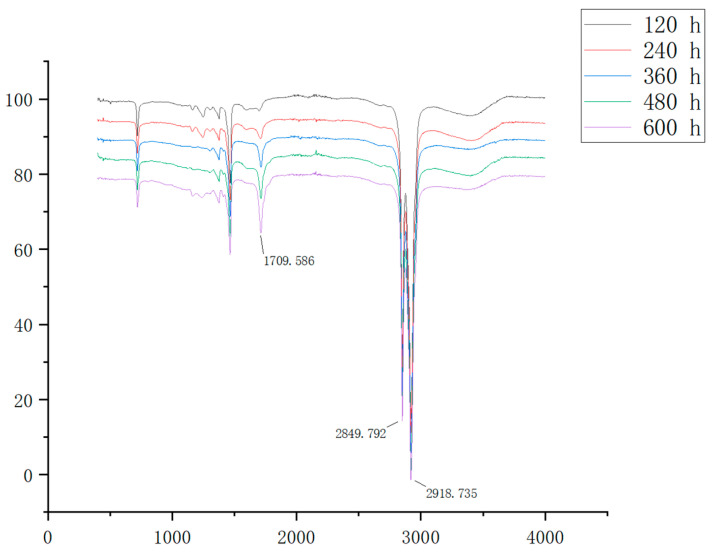
Infrared spectrum of the POE film after thermal oxidative aging.

To evaluate the potential formation of carbonyl compounds during the aging process of POE films, infrared spectroscopy was employed to analyze the POE film samples before and after aging, as illustrated in Figure 10. Specifically, the absorption peak at 2920 cm^−1^ corresponds to the stretching vibration of tertiary carbon (C-H) bonds, while the peak at 2850 cm^−1^ represents the stretching vibration of methyl groups (C-H) bonds, and the peak at 1710 cm^−1^ indicates the stretching vibration of carbonyl groups. 

**Figure 11:**
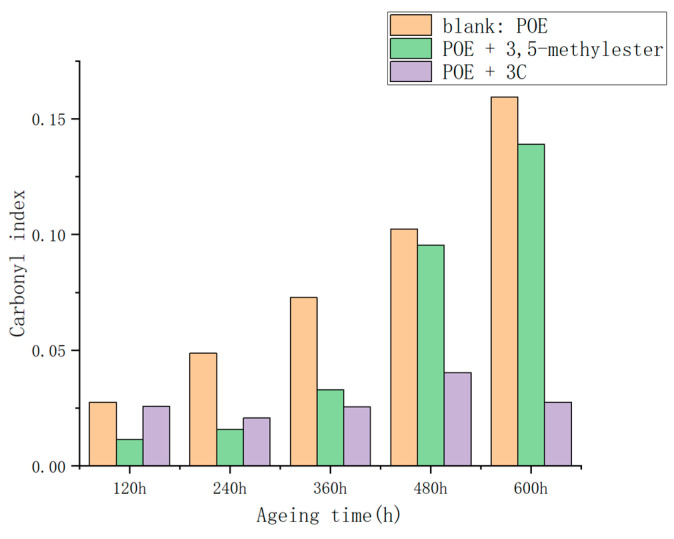
Carbonyl index in POE films after thermal oxidative aging.

The degree of aging of the POE films was assessed using the carbonyl index, calculated as the ratio of the absorption area of the carbonyl peak (1710 cm^−1^) to that of the internal standard peak, unaffected by photoaging. In this study, the peak at 2920 cm^−1^ was selected as the internal standard peak. The carbonyl index results for the POE film samples before and after aging are presented in Figure 11.

The carbonyl index of the POE films containing **3C** exhibited a gradual change, whereas that of the blank and 3,5-methylester groups of POE films increased rapidly with aging time. This suggests that **3C** can effectively inhibit the oxidation process and reduce the formation of carbonyl compounds.

#### 3.2.6. Light Transmittance Measurement of POE Films after Ultraviolet Aging

**Figure 12:**
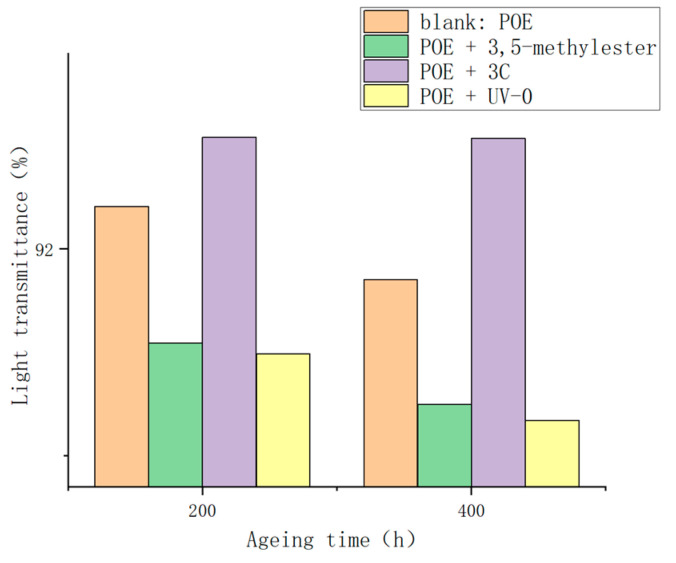
Light transmittance in POE films after ultraviolet aging.

As the POE films undergo aging within the ultraviolet aging chamber, the transmittance of each group exhibits a declining trend. It is evident from Figure 12 that POE films with added **3C** experience the least reduction in transmittance after ultraviolet aging, with only a 0.06% decrease. Conversely, the transmittance reduction trends of the other three groups (blank: POE, POE + 3,5-methylester, POE + UV-0) surpass that of POE with added **3C**. This indicates that the homemade anti-aging agent 3C effectively inhibits the formation of chromophoric groups. The result that POE films containing UV-0 exhibit relatively low light transmittance is somewhat unexpected. This could be attributed to the deep yellow color of UV-0 itself, which consequently affects the light transmittance of the film. In fact, several factors influence the light transmittance of POE films. Besides the chromophoric groups produced by the aging of POE, the color contamination of the additives themselves, particularly secondary aromatic amine antioxidants, can impact the light transmittance of POE.

#### 3.2.7. Carbonyl Index Determination in POE Film after Ultraviolet Aging

The infrared spectroscopic analysis of POE film samples before and after exposure to ultraviolet (UV) radiation was conducted. The absorption peak observed at 2920 cm^−1^ corresponds to the stretching vibration of tertiary carbon (C-H) bonds, while the peak at 2847 cm^−1^ corresponds to the stretching vibration of methyl (C-H) bonds, and the peak at 1710 cm^−1^ corresponds to the stretching vibration of carbonyl groups.

**Figure 13:**
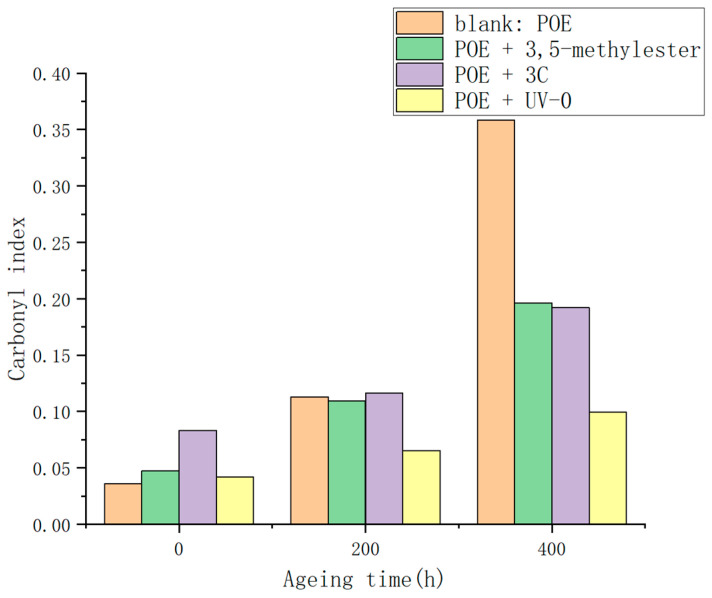
Carbonyl index in POE films after ultraviolet aging.

By specifically analyzing the carbonyl stretching vibration peak at 1710 cm^−1^ and the absorption peak at 2920 cm^−1^, the carbonyl index of each sample after ultraviolet (UV) aging was calculated, with the results depicted in Figure 13.

It is noteworthy that the blank group, which was not treated with any anti-aging agents, exhibited a significant increase in the carbonyl index after undergoing two stages of UV irradiation, rising from 0.036 to 0.359. Conversely, a group of samples treated with 2,4-dihydroxybenzophenone, a precursor for synthesizing the anti-aging compound **3C**, showed minimal increase in the carbonyl index after 400 h of UV aging. Similarly, the group of samples treated with the homemade anti-aging agent **3C** demonstrated a relatively small increase in the carbonyl index, indicating the role of the hydroxybenzophenone structural moiety within **3C** in mitigating UV-induced aging effects and reducing the formation of carbonyl compounds.

## 4. Conclusions

A hindered phenol-linked benzophenone hybrid compound **3C** as a novel synthetic polymer anti-aging agent with a definite chemical structure was synthesized and characterized. Its thermal stability and UV resistance were assessed. Subsequently, the compound **3C** as an additive was incorporated into the preparation of polyolefin elastomer (POE) films and evaluated for its effect on the anti-aging performance of POE film. The results indicate that the compound **3C** combined the thermal-oxidative aging resistance of hindered phenol with the ultraviolet aging resistance of benzophenone exhibiting anti-aging performance in both thermo-oxidative aging and ultraviolet aging tests of POE films, and can become a potential multifunctional polymer additive with good anti-aging performance. These results open new perspectives toward the development of synthetic multifunctional polymer additive for application as polymer anti-aging agents in functional materials. However, when 3C is specifically applied in fields such as cosmetics and food packaging, comprehensive pharmacological and toxicological studies are necessary to thoroughly assess its safety in future.

## Figures and Tables

**Table 1:** Melt flow index of POE pellets after extrusion.

	Blank: POE	POE + 3,5-Methylester	POE + 3C	POE + UV-0
Melt flow index	4.80 g/10 min	4.64 g/10 min	4.00 g/10 min	4.84 g/10 min

## Data Availability

Data are contained within the article.
